# Cryo-instrumentation technique: a temperature-modulated approach to improve the mechanical performance of NiTi endodontic instruments

**DOI:** 10.1016/j.jobcr.2026.101519

**Published:** 2026-08-04

**Authors:** Ajay Singh Rao, Ajinkya M. Pawar, M. Ashwin Surana, Riddhi Vasa, Shreya Volety, Unmesh Khanvilkar, Dian Agustin Wahjuningrum, Norhana Jusoh

**Affiliations:** a Department of Conservative Dentistry & Endodontics, K M Shah Dental College & Hospital, Sumandeep Vidyapeeth deemed to be University, Vadodara, Gujarat, India; b Department of Conservative Dentistry and Endodontics, Nair Hospital Dental College, Affiliated to Maharashtra University of Health Sciences (MUHS), Mumbai, Maharashtra, 400008, India; c Department of Conservative Dentistry and Endodontics, Bharati Vidyapeeth Dental College, Navi Mumbai, Maharashtra, India; d Department of Conservative Dentistry, Faculty of Dental Medicine, Universitas Airlangga, Surabaya, Indonesia; e Department of Biomedical Engineering and Health Sciences, Faculty of Electrical Engineering, Universiti Teknologi Malaysia (UTM), Johor Bahru, Johor, 81310, Malaysia; f Medical Device Technology Center (MEDiTEC), Institute Human Centred Engineering (iHumEn), Universiti Teknologi Malaysia (UTM), Johor Bahru, Johor, 81310, Malaysia

**Keywords:** Broken instrument, Canal centering ability, Cryo-instrumentation, Cyclic fatigue resistance, File separation, Intracanal temperature

## Abstract

**Background:**

The mechanical behavior and shaping performance of heat-treated nickel–titanium (NiTi) rotary instruments are influenced by temperature-dependent phase transformation.

**Aim:**

This study evaluated cryo-instrumentation technique, in which NiTi instruments are used under controlled cryogenic conditions, and assessed its effect on cyclic fatigue resistance, cutting efficiency, and canal centering ability of HyFlex CM instruments.

**Materials and methods:**

Thirty-six samples were randomly allocated into two groups. In the cryo-instrumentation group, HyFlex CM instruments were surface-cooled with Endo-Frost® and used under intracanal cryogenic conditions at 2°C. In the body-temperature group, instruments were used at 37°C without surface cooling. Cyclic fatigue resistance was assessed using standardized artificial canals, and the number of cycles to fracture was calculated. Cutting efficiency was evaluated by measuring dentin reduction on pre- and post-instrumentation cone-beam computed tomography scans. Canal centering ability was assessed using standardized two-dimensional image superimposition at the coronal, middle, and apical thirds. Intergroup comparisons were performed with statistical significance set at p < 0.05.

**Results:**

Instruments used under cryogenic conditions demonstrated significantly greater cyclic fatigue resistance than those used at body temperature (p < 0.001). Cutting efficiency did not differ significantly between the groups at any canal level (p > 0.05). Canal centering ability was significantly improved in the cryo-instrumentation group at the middle and apical thirds (p < 0.05), whereas no significant difference was observed at the coronal third.

**Conclusion:**

The proposed cryo-instrumentation technique may enhance cyclic fatigue resistance and canal centering ability of HyFlex CM instruments without compromising cutting efficiency.

## 1 Introduction

Instrument separation is one of the most common and clinically challenging complications faced in root canal treatment, frequently undermining procedural effectiveness and overall treatment results. Among the numerous factors leading to instrument breakage, cyclic fatigue and torsional stress are commonly seen as the primary mechanisms.^1^ Therefore, significant research initiatives have concentrated on enhancing the cyclic fatigue resistance (CFR) of nickel–titanium (NiTi) endodontic instruments. Innovations in NiTi instrument technology have mainly revolved around thermomechanical heat-treatment methods aimed at altering the alloy phase composition. These procedures elevate the ratio of the martensitic (M) phase, which is fundamentally influenced by temperature and demonstrates enhanced flexibility along with improved resistance to cyclic fatigue relative to the austenitic phase.^2^ In low temperature environments, NiTi instruments generally retain a majority in the martensitic (M) form, since it is the ‘low temperature’ phase.^3^ This trait is beneficial for instrumentation in the curved canals. Literature indicates that devices with increased martensitic content exhibit improved fatigue resistance, highlighting the focus on this phase in modern heat-treated NiTi systems.^4^, ^5^, ^6^ The HyFlex CM tools (Coltene Whaledent, Switzerland), produced with controlled memory technology, illustrate these metallurgical innovations. These files are claimed to have greater flexibility, diminished shape-memory rebound, and enhanced cyclic fatigue resistance compared to standard NiTi files, features mainly due to their mainly martensitic microstructure.^7^ Nonetheless, instrument fracture still happens in clinical situations, especially when procedures are carried out at body temperature.^8^

The impact of environmental and intracanal temperature on the mechanical properties of NiTi instruments has gained significant recognition. Earlier research has shown that reduced ambient temperatures greatly improve the cyclic fatigue resistance of NiTi rotary files, while rising temperatures from about 4°C to 35°C or more lead to a significant decline in fatigue resistance.^9^^,^^10^ This temperature-related decline has been noted in both traditional NiTi instruments and in newer heat-treated systems, such as HyFlex CM files.^11^^,^^12^

Cryotherapy (cold saline) has been integrated into endodontic practice mainly for its biological advantages, such as reducing postoperative pain and inflammation while preserving hard-tissue integrity through vasoconstriction.^13^^,^^14^ The application of cold saline (at 2°C) irrigation has been found to effectively lower intracanal temperature and sustain this temperature reduction for several minutes.^15^ Endo-Frost® (Coltene Whaledent, Switzerland) has been used clinically to transiently reduce the surface temperature of heat-treated nickel titanium instruments; such cooling is known to favor martensitic (M) phase stabilization, which helps to maintain a pre-curved shape of the ‘heat-treated’ files during canal preparation.^2^^,^^16^

While the impacts of heat treatment, intracanal temperature, cryotherapy, and instrument surface cooling have been studied separately, existing endodontic procedures do not include a uniform chairside method that intentionally keeps a lowered intracanal temperature during the instrumentation in the canal to enhance the metallurgical performance of heat-treated NiTi instruments. This disparity justified the creation and suggestion of an innovative temperature-modulated instrumentation method, proposed as the ‘cryo-instrumentation’ technique, which integrates cooling of instrument surfaces with regulated intracanal cryogenic irrigation throughout the canal preparation.

The null hypothesis was postulated that utilizing the proposed ‘cryo-instrumentation’ technique would not lead to a statistically significant difference in cyclic fatigue resistance, cutting efficiency, or canal centering ability of heat-treated nickel–titanium endodontic instruments compared to instrumentation performed at body temperature (37°C) without any surface treatment.

## 2 Methods

### 2.1 Study design and reporting guidelines

**Fig. 1:**
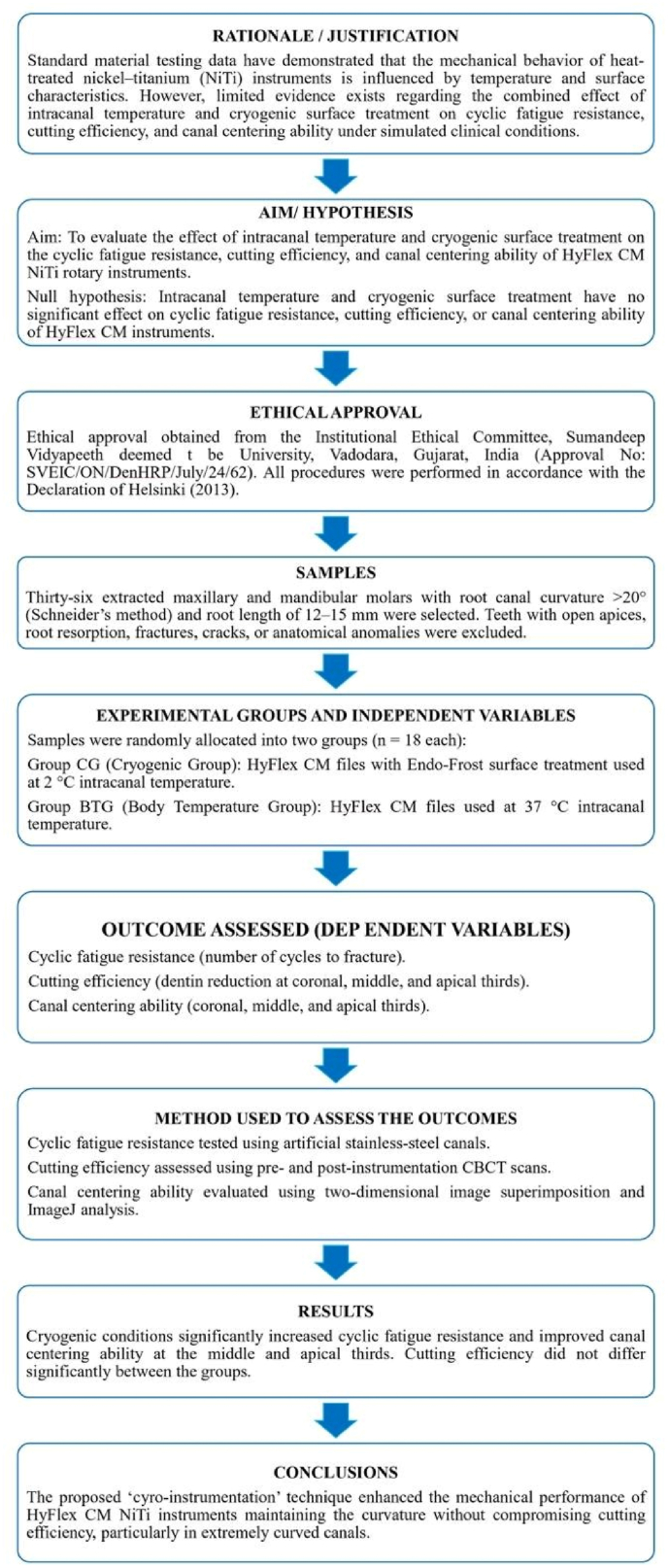
PRILE 2021 flow diagram.

This laboratory-based experimental study was designed and reported in accordance with the Preferred Reporting Items for Laboratory Studies in Endodontology (PRILE) 2021 guidelines (Fig. 1).

**Storage of specimens:** Following extraction, all teeth were cleaned to remove adherent soft tissue remnants and calculus using an ultrasonic scaler. The specimens were then disinfected and stored in **0.1% thymol solution at room temperature** until experimentation. Before use, the teeth were thoroughly rinsed with distilled water to remove any residual thymol and subsequently maintained in **normal saline** to prevent dehydration until the experimental procedures were performed.

**Temperature monitoring**: The temperature of the saline bath was monitored continuously throughout the cyclic fatigue experiment using a calibrated digital thermometer (Hanna Instruments India Pvt. Ltd., Mumbai, India; accuracy: ±0.2°C; resolution: 0.1°C). The temperature probe was positioned immediately adjacent to the artificial canal without contacting the rotating instrument or the stainless-steel block. For the cryo-instrumentation group, the saline bath was maintained at 2 ± 1°C, whereas for the body-temperature group it was maintained at 37 ± 1°C. Temperature was verified before insertion of each instrument and continuously observed during rotation. When the temperature deviated beyond the predefined range, testing was temporarily discontinued until the target temperature was re-established. Fresh refrigerated or warmed saline was added as required to maintain the designated experimental temperature. The temperature recorded by the probe represented the temperature of the saline surrounding the artificial canal rather than a direct measurement from within the canal lumen.

For instrumentation of the extracted teeth, saline used for irrigation was maintained at 2°C or 37°C, according to group allocation, in separate temperature-controlled containers. The irrigant temperature was verified immediately before delivery using the same calibrated thermometer. Fresh irrigant was replenished throughout instrumentation to minimize temperature equilibration with the surrounding environment. However, intracanal temperature was not directly measured within the extracted teeth; therefore, the reported temperatures represent the temperature of the irrigant immediately before delivery rather than confirmation that the canal remained continuously at the target temperature.

### 2.2 Ethical approval

Ethical approval was obtained from the Institutional Ethics Committee of Sumandeep Vidyapeeth (Deemed to be University), Vadodara, Gujarat, India (approval number: SVEIC/ON/DenHRP/feb/25/54). All procedures were conducted in accordance with applicable institutional and ethical guidelines.

### 2.3 Sample size calculation

Sample size estimation was performed using G*Power software (version 3.1). Assuming a medium effect size (0.5), an alpha error probability of 5%, and a statistical power of 80%, a minimum sample size of 18 specimens per group was determined.

### 2.4 Sample selection

**Fig. 2:**
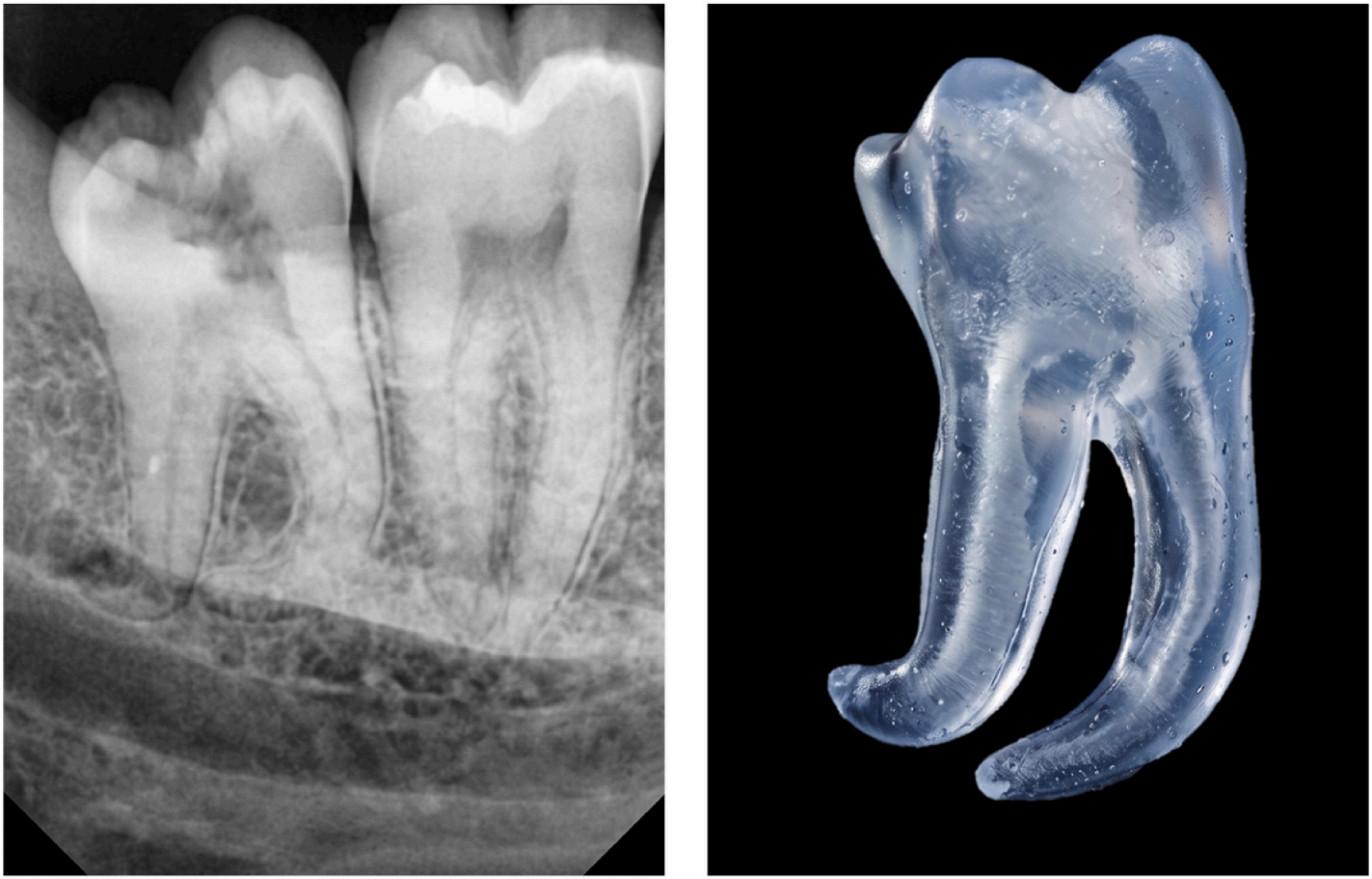
Specimen selection and experimental models Representative radiograph of a severely curved mandibular molar selected for the study (left) and a transparent resin tooth model with comparable root canal curvature used for standardized canal centering assessment (right).

Extracted human maxillary and mandibular molars indicated for extraction due to periodontal reasons or extensive carious involvement were included. Teeth exhibiting root canal curvature greater than 20°, as determined using Schneider's method,^9^ were selected. Teeth with open apices, root fractures, internal or external resorption, visible cracks, calcifications, or anatomical anomalies were excluded. Representative radiographic confirmation of severe canal curvature and the corresponding experimental resin model are shown in Fig. 2.

### 2.5 Randomization and group allocation

Specimens were randomly allocated using a computer-generated randomization sequence (www.randomizer.org). Allocation concealment was ensured using opaque sealed envelopes. The samples were equally divided into two experimental groups (n = 18 per group):

• **Group CG (Cryo-instrumentation group):** HyFlex CM nickel–titanium files (Coltene Whaledent, Switzerland) were used under cryogenic intracanal conditions (2°C) following surface treatment with Endo-Frost® (Coltene Whaledent, Switzerland)
• **Group BTG (Body temperature group):** HyFlex CM NiTi files (Coltene Whaledent, Switzerland) used under body temperature conditions (37°C) without Endo-Frost® surface treatment.

The following parameters were evaluated: cyclic fatigue resistance (CFR), cutting efficiency (CE), and canal centering ability (CCA).

### 2.6 Cyclic fatigue resistance

Cyclic fatigue resistance was evaluated using artificial canals prepared in a stainless-steel block, following previously established methodologies. The simulated canal exhibited a curvature angle of 90°, a radius of curvature of 5 mm, and a width of 1.5 mm. To allow free rotation and minimize instrument binding, the canal diameter was enlarged by 0.2 mm relative to the instrument size. The artificial canals were precisely milled using a computer-assisted diode laser system.

A torque-controlled endomotor with a 20:1 reduction handpiece was used to run pre-curved HyFlex CM nickel-titanium instruments (size 20/04) at 500 rpm with a torque of 2.5 Ncm. To lessen friction between the instrument and the canal walls, a high-flow synthetic lubricant (Super Oil; Singer Co. Ltd., Elizabeth, NJ, USA) was used. To provide consistent straight-line access, every instrument was positioned at 0°.

**Fig. 3:**
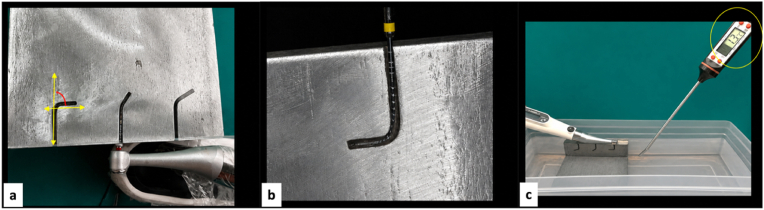
Experimental setup for cyclic fatigue resistance testing. (a, b) Stainless-steel block containing a simulated artificial canal with a 90° curvature and a 5-mm radius of curvature used for cyclic fatigue testing and (c) Temperature-controlled saline bath employed during testing, with real-time monitoring of intracanal temperature using a digital thermometer to maintain either cryogenic (2°C) or body-temperature (37°C) conditions. Instrument fracture was detected visually and/or audibly, and the time to fracture was recorded using a digital stopwatch. The number of cycles to fracture (NCF) was calculated using the formula: NCF = (time to fracture × rotational speed)/60.

Fig. 3 a and b show the temperature-control setup and cyclic fatigue testing assembly. Files in Group BTG were rotated in a saline bath (0.9% sodium chloride; Aculife Healthcare Pvt. Ltd., Ahmedabad, India) kept at 37°C, whereas Endo-Frost®-treated instruments in Group CG were revolved in a saline bath kept at 2°C. A calibrated digital thermometer (Hanna Instruments India Pvt. Ltd., Mumbai, India) placed next to the artificial canal was used to continuously measure intracanal temperature (Fig. 3c).

### 2.7 Cutting efficiency

Cutting efficiency was assessed by measuring dentin reduction at the cervical, middle, and apical thirds using pre- and post-instrumentation cone-beam computed tomography (CBCT) scans. To guarantee consistent placement during imaging, teeth with extreme root curvature were placed in extra silicone molds. A CBCT equipment (Carestream CS 9300, Carestream Dental, France) with defined exposure parameters (11 × 8 cm field of view, 90 kVp, 10 mA, voxel size 150 μm) was used to generate preoperative CBCT scans. Every scan was carried out in a lab setting. Endo Access burs were used to prepare the access cavity, and Endo-Z burs (Dentsply Sirona, USA) were used for deroofing. Working length was calculated radiographically using Ingle's method, and canal patency was established using ISO #10 K-files (Mani, Japan). Using ISO #10 K-files or reamers (Mani, Japan) and EDTA gel (Glyde, Dentsply, USA), the glide path was prepared.

**Fig. 4:**
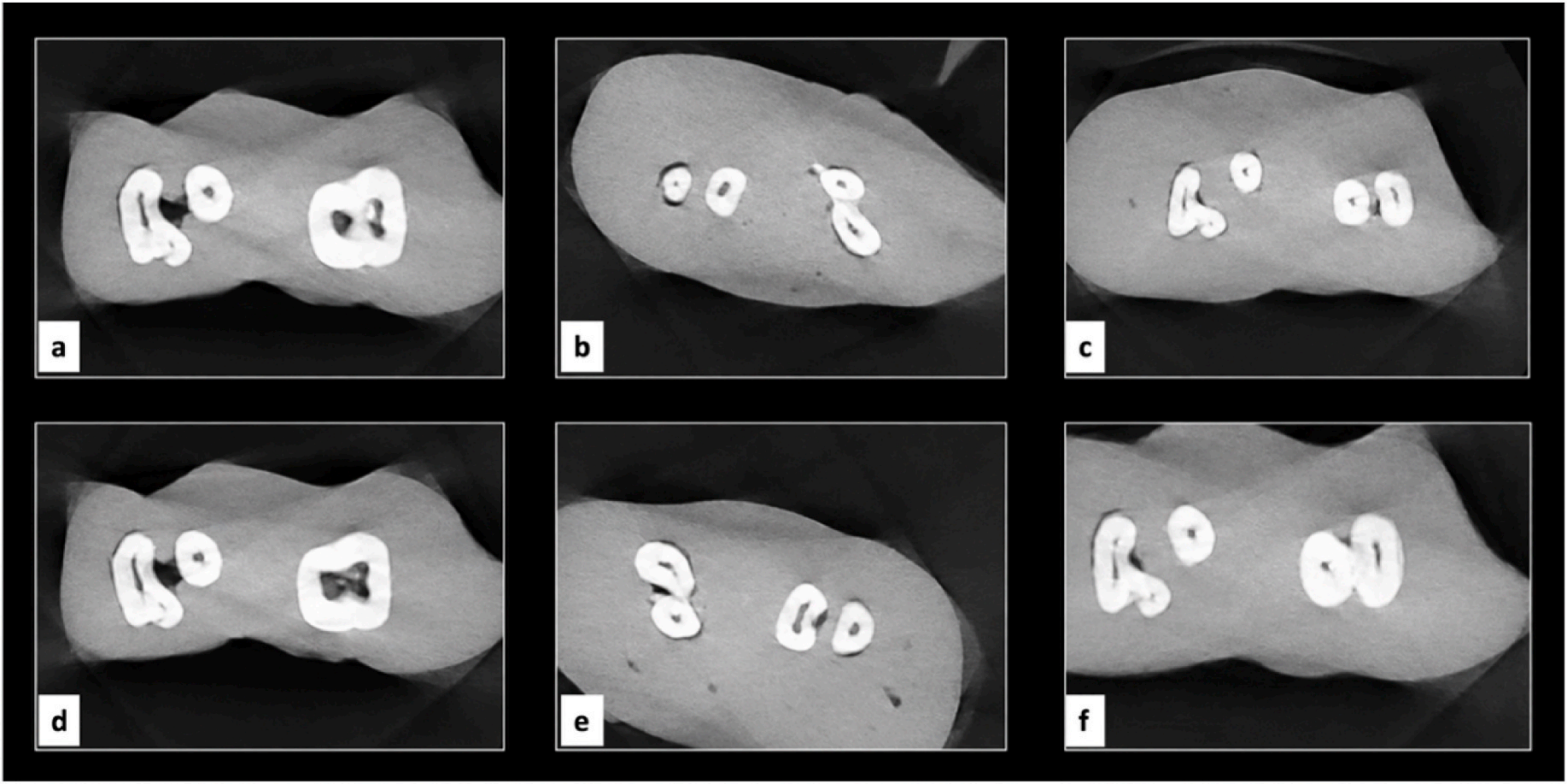
Representative CBCT images used for cutting efficiency analysis Axial cone-beam computed tomography (CBCT) sections obtained before (a–c) and after (d–f) canal instrumentation, demonstrating dentin removal at the cervical, middle, and apical thirds for the assessment of cutting efficiency. CBCT measurements were independently performed by two experienced observers who were blinded to the experimental group allocation.

HyFlex CM files with Endo-Frost® surface treatment and continuous irrigation with cold saline (2°C) administered via a 27-gauge syringe were used to prepare the canals in Group CG. Canal preparation in Group BTG was performed using HyFlex CM nickel–titanium files (Coltene Whaledent, Switzerland) with continuous irrigation using warm saline maintained at body temperature (37°C) (0.9% sodium chloride; Aculife Healthcare Pvt. Ltd., Ahmedabad, India), delivered through a 27-gauge flexible, side-vented irrigation needle (NaviTip®, Ultradent Products Inc., South Jordan, UT, USA), without surface treatment of the instruments. In both groups, instrumentation was standardized to size 20/04. The same imaging parameters were used to produce post-instrumentation CBCT scans. By comparing pre- and post-instrumentation scans in the cervical, middle, and apical thirds in axial, sagittal, and coronal sections, dentin thickness measurements were made using NNT software (NewTom NNT, QR srl, Verona, Italy). Fig. 4 displays representative CBCT sections utilized for cutting efficiency analysis.

### 2.8 Canal centering ability

#### 2.8.1 Specimen preparation

Transparent, high-quality resin teeth with severe root curvature were used to simulate clinically relevant canal anatomy while allowing standardized visualization of shaping behavior (Fig. 4). Canal patency, working length determination, and glide path preparation were performed as described previously. All instrumentation procedures were carried out by a single operator under a dental operating microscope (Magna, Labomed, Los Angeles, USA). Specimens were randomly allocated to Group CG and Group BTG (n = 18 per group), and canal preparation was performed using the same protocol described for cutting efficiency.

#### 2.8.2 Image acquisition and analysis

Each specimen was securely positioned on a fixed platform and photographed using a digital camera (Canon, Tokyo, Japan) equipped with a macro lens (Canon, EF 100 mm, f/2.8L IS USM). Standardized digital images were obtained before and after instrumentation.

Slicer software (version 5.0.3) was used to co-register pre- and post-instrumentation pictures utilizing a strict registration approach based on image intensity similarity. Adobe Photoshop (CS6; Adobe Systems, San Jose, CA, USA) was then used to superimpose the registered photos with a standard image resolution of 900 × 2352 pixels. ImageJ software (National Institutes of Health, Bethesda, MD, USA) was used to measure canal centering deviation.

Images were orientated horizontally for consistency, with the apical aspect on the right and the coronal aspect on the left. The coronal, middle, and apical thirds were measured at three predetermined locations along the X-axis (784, 1568, and 2352 pixels). Two calibrated endodontists who were blind to group assignment carried out each measurement on their own.

**Fig. 5:**
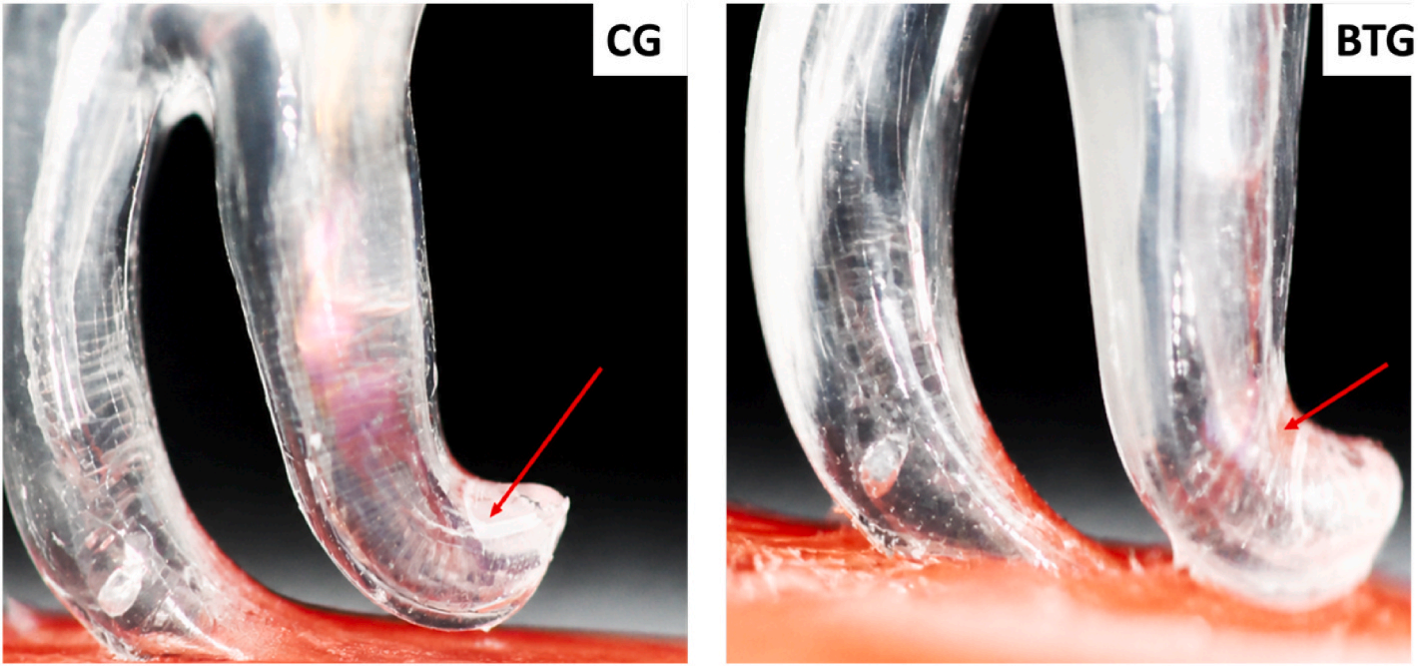
Representative qualitative observation of file behavior in transparent models (a) Group CG: Under cryogenic conditions, the instrument maintains its pre-curved configuration and closely follows the canal curvature in the apical third (red arrow). (b) Group BTG: Under body-temperature conditions, the instrument exhibits a tendency to straighten and deviate from the canal path (red arrow).

In addition to quantitative assessment, representative visual observations of file behavior during canal preparation were recorded. Under cryogenic conditions, instruments maintained their pre-curved configuration and followed the canal curvature more closely in the apical third, whereas instruments used at body temperature exhibited a tendency to straighten and deviate from the canal path (Fig. 5). Because canal-centering ability was assessed from two-dimensional images, the measurements represented canal displacement within the standardized imaging plane at the three predefined canal levels and did not quantify three-dimensional transportation.

#### 2.8.3 Blinding

All experimental procedures were performed by a single operator. Due to the nature of the intervention, operator blinding was not feasible; however, outcome assessment and image analysis were conducted by two blinded evaluators.

#### 2.8.4 Statistical analysis

SPSS software (version 30.0; IBM Corp., Armonk, NY, USA) was used for statistical analysis. The mean ± standard deviation was used to express the data. The Shapiro-Wilk test was used to determine whether the data distribution was normal. Independent samples t-tests were used for intergroup comparisons, and one-way analysis of variance (ANOVA) and, if necessary, Tukey's post hoc test were used for intragroup comparisons across canal levels. The intraclass correlation coefficient (two-way random-effects model, absolute agreement) was used to assess interobserver reliability for canal centering measurements. P < 0.05 was chosen as the threshold for statistical significance.

## 3 Results

Data were normally distributed as confirmed by the Shapiro–Wilk test, and parametric statistical analyses were applied. Results are presented as mean ± standard deviation.

## 4 Cyclic fatigue resistance

**Fig. 6:**
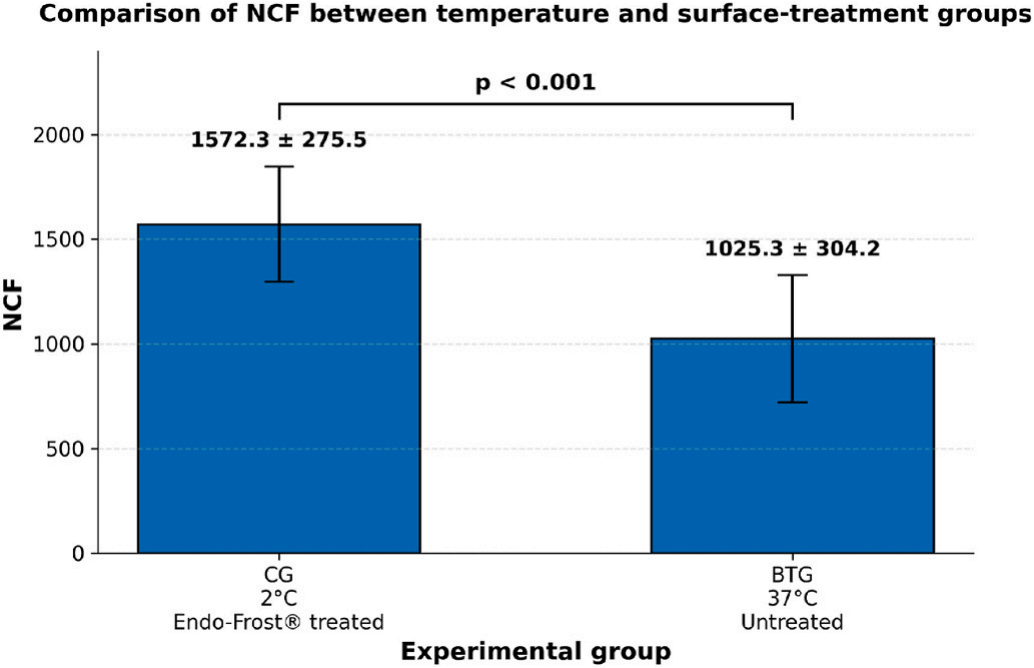
Comparison of nickel-titanium cyclic fatigue resistance between the cold-treated control group and body-temperature group.

Group CG demonstrated significantly higher cyclic fatigue resistance compared with Group BTG. Instruments operated under cryogenic conditions exhibited a greater number of cycles to fracture than those used at body temperature (37°C), and this difference was statistically significant (*p* < 0.001) (Fig. 6). The means, standard deviations, confidence intervals, and exact p value is presented in Table 1.

**Table 1:** Comparison of cyclic fatigue resistance between the cryo-instrumentation group (CG) and body-temperature group (BTG).

Group	Temperature Condition	Surface Treatment	NCF (Mean ± SD)	95% CI	Exact *P* value
Group CG	2°C	Endo-Frost® treated	1572.3 ± 275.5	1435.3–1709.3	<0.001
Group BTG	37°C	Untreated	1025.3 ± 304.2	874.1–1176.5	—

## 5 Cutting efficiency

**Fig. 7:**
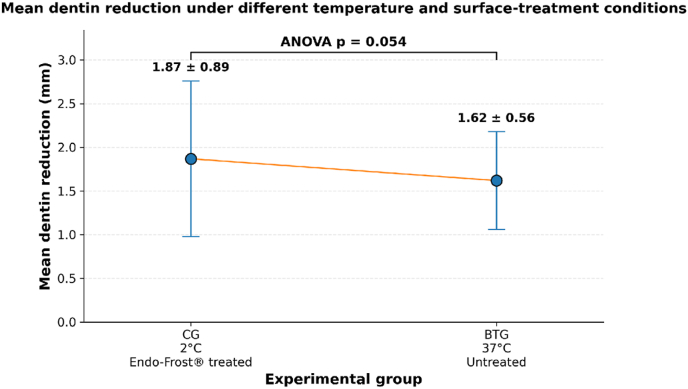
Dot plot comparing mean dentin reduction between the cold-treated control group and body-temperature group.

No statistically significant difference in cutting efficiency was observed between Group CG and Group BTG at the coronal, middle, or apical thirds (*p* > 0.05). Dentin reduction values were comparable between the two groups across all canal levels (Fig. 7). The means, standard deviations, confidence intervals, and exact p value is presented in Table 2.

**Table 2:** Comparison of cutting efficiency between the cryo-instrumentation group (CG) and body-temperature group (BTG).

Group	Temperature Condition	Surface Treatment	Mean Dentin Reduction (mm ± SD)	95% CI	Exact *P* value
**Group CG**	2°C	Endo-Frost® treated	1.87 ± 0.89	1.43–2.31	0.054
**Group BTG**	37°C	Untreated	1.62 ± 0.56	1.34–1.90	—

## 6 Canal centering ability

**Fig. 8:**
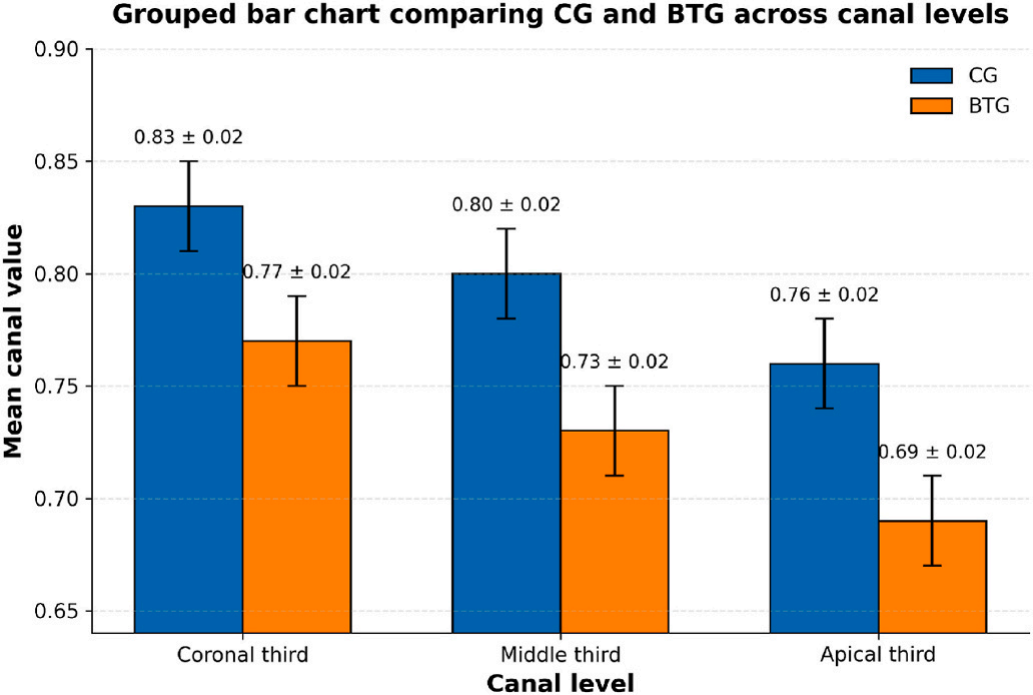
Grouped bar chart comparing the mean canal values of the cold-treated control group (CG) and body-temperature group (BTG) at the coronal, middle, and apical thirds.

Intergroup comparison revealed no statistically significant difference in canal centering ability between Group CG and Group BTG at the coronal third (*p* > 0.05). However, statistically significant differences were observed at the middle and apical thirds, with Group CG demonstrating superior centering ability compared with Group BTG (*p* < 0.05) (Fig. 8). Intragroup analysis showed a progressive reduction in canal centering ability from the coronal to the apical third within both Group CG and Group BTG, and these differences were statistically significant (*p* < 0.05). Interobserver agreement for canal centering measurements demonstrated high reliability, as indicated by a strong intraclass correlation coefficient. The means, standard deviations, confidence intervals, and exact p value is presented in Table 3.

**Table 3:** Comparison of canal centering ability between the cryo-instrumentation group (CG) and body-temperature group (BTG).

Canal Level	Group CG (Mean ± SD)	95% CI	Group BTG (Mean ± SD)	95% CI	Exact *P* value
**Coronal third**	0.83 ± 0.02	0.82–0.84	0.77 ± 0.02	0.76–0.78	0.176
**Middle third**	0.80 ± 0.02	0.79–0.81	0.73 ± 0.02	0.72–0.74	0.012
**Apical third**	0.76 ± 0.02	0.75–0.77	0.69 ± 0.02	0.68–0.70	<0.001

## 7 Discussion

The current in vitro investigation assessed how the mechanical performance and shaping behavior of HyFlex CM nickel-titanium rotary instruments were affected by intracanal temperature modulation in conjunction with Endo-Frost® surface cooling. The results show that temperature has a substantial impact on the behavior of the instrument; cutting efficiency was unchanged, but cycle fatigue resistance and canal centering ability showed statistically significant changes. The null hypothesis was thus partially rejected.

Instruments operating in cryogenic conditions showed a significant improvement in cycle fatigue resistance. Fig. 6 illustrates that, in comparison to Group BTG, Group CG showed a noticeably greater number of cycles to fracture. This behavior is consistent with the results of the previous studies that showed that the temperature-dependent phase transformation characteristics of NiTi alloys, in which reduced temperatures favor martensitic stabilization and improved fatigue resistance under cyclic loading conditions.^17^, ^18^, ^19^ On the other hand, exposure to body warmth encourages transition into the stiffer austenitic phase, which lowers fatigue resistance. The literature has repeatedly documented similar temperature-related effects on the cycle fatigue resistance of nickel-titanium instruments.^20^^,^^21^ These findings provide validity to the theory that, under repeated cyclic stress, maintaining a low-temperature environment during instrumentation may postpone fracture development and propagation.

Although fatigue resistance was improved, intracanal temperature had little effect on cutting efficiency. Dentin removal results were similar between Group CG and Group BTG at all canal levels, as Fig. 7 illustrates. This result implies that design-related factors, such as cross-sectional geometry, rake angle, and taper, rather than just thermal conditions, largely control the cutting performance of HyFlex CM instruments. Previous investigations assessing the cutting efficiency of heat-treated nickel-titanium instruments under various experimental conditions have shown similar findings.^22^, ^23^, ^24^ These findings are significant from a therapeutic standpoint because they show that dentin-cutting ability is not compromised by the mechanical benefits of cryogenic treatment.

In terms of canal centering ability, cryogenic instruments performed better in the middle and apical thirds, but there was no statistically significant difference at the coronal third. In the central and apical regions, Group CG had noticeably superior centering ability compared to Group BTG, as seen in Fig. 8. Because of their anatomical complexity, these curved canal sections are especially vulnerable to canal transit.^25^ In certain areas, better adaptation to canal curvature may be possible due to the instruments' higher flexibility at lower temperatures, which could lead to improved centering behavior. Similar findings have been documented in previous studies evaluating shaping results in curved canals.^26^, ^27^, ^28^ Because of the comparatively straighter architecture and bigger canal width in this area, where instrument flexibility is less important, there may not be a noticeable change at the coronal third.

A standardized two-dimensional image-superimposition method was used to assess canal-centering ability in the transparent resin models. This approach permitted direct visualization of instrument behaviour and enabled measurements at reproducible, predefined canal levels under standardized imaging conditions. Rigid co-registration of the pre- and post-instrumentation images, fixed specimen positioning, predetermined measurement coordinates, and independent assessment by two blinded evaluators were used to improve measurement reproducibility.

Nevertheless, micro-computed tomography is regarded as a more comprehensive method for evaluating root-canal shaping because it permits non-destructive three-dimensional comparison of the canal anatomy before and after instrumentation. Micro-CT can quantify canal transportation and centering ability in multiple planes and throughout the canal volume, while also assessing changes in canal volume, surface area, dentin removal, and unprepared canal walls. In contrast, the two-dimensional method used in the present study evaluated displacement only within the selected imaging plane and at three predetermined measurement locations. Consequently, transportation occurring in the buccolingual dimension or between the selected measurement levels may not have been detected.

The use of image registration and manual identification of canal boundaries may also have introduced measurement bias arising from minor differences in specimen orientation, image magnification, pixel calibration, superimposition accuracy, and examiner-dependent landmark selection. Although standardized positioning, rigid image registration, examiner calibration, blinding, and duplicate measurements were used to reduce these sources of error, they cannot be eliminated completely. Therefore, the observed differences in canal-centering ability should be interpreted as two-dimensional estimates rather than a complete three-dimensional representation of canal transportation. Future studies should validate these findings using high-resolution micro-CT analysis of extracted teeth.

The current study's findings are consistent with earlier research showing that heat-treated nickel-titanium instruments' resilience to cycle fatigue is negatively impacted by rising temperatures.^18^^,^^29^ Furthermore, it has been demonstrated that intracanal cryotherapy has positive biological effects without adversely affecting endodontic operations, especially in lowering postoperative pain and inflammation.^30^ A systematic review further confirmed that body temperature (37°C) significantly reduces the cyclic fatigue resistance of both conventional and newer-generation nickel-titanium instruments.^31^ Collectively, these findings provide strong mechanistic support for the rationale underlying the proposed cryo-instrumentation technique.

Although maintaining a constant intracanal temperature of approximately 2°C throughout instrumentation may be challenging under routine clinical conditions, previous in vivo investigations by de Hemptinne et al.^33^ demonstrated that intracanal irrigants undergo progressive thermal equilibration rather than instantaneous warming. Their findings showed that substantial thermal equilibration toward body temperature occurs over approximately the first 10 s following intracanal delivery, providing a brief interval during which the irrigant remains appreciably cooler than the surrounding tissues. Considering that rotary instrumentation is typically performed in short cycles of approximately 3–4 s before withdrawal of the instrument for cleaning and replenishment with fresh cooled irrigant, repeated irrigation may help sustain a transiently reduced intracanal temperature during active instrumentation. Consequently, despite the progressive warming of the irrigant, the proposed cryo-instrumentation protocol may permit maintenance of a cooler intracanal environment during successive instrumentation cycles. Nevertheless, the present study should be regarded as a laboratory proof-of-concept investigation, and further clinical studies are warranted to evaluate the practicality, operator feasibility, patient comfort, additional chairside time, and overall clinical applicability of the proposed cryo-instrumentation technique.

Although micro-computed tomography is considered the reference standard for three-dimensional assessment of canal shaping, the present study evaluated canal centering ability using transparent resin models and a standardized image superimposition technique, which has been widely adopted in experimental endodontic research because of its reproducibility and direct visualization of instrument behavior. Standardized specimen positioning, rigid image registration, predetermined measurement sites, and blinded evaluators were employed to minimize measurement bias. Nevertheless, because this method evaluates canal centering in a two-dimensional plane, it cannot fully characterize three-dimensional canal transportation, which remains a limitation of the present study.

Within the limitations of this laboratory-based study, cryo-instrumentation using combined Endo-Frost surface cooling and intracanal irrigation at 2°C improved the cyclic fatigue resistance and canal-centering ability of HyFlex CM instruments without significantly affecting cutting efficiency. These findings should be regarded as preliminary and specific to the experimental conditions, instrument system, and assessment methods used. Further preclinical and clinical studies are required to determine the reproducibility, safety, practicality, patient acceptability, and clinical relevance of this protocol before its routine use can be considered.

The individual contribution of Endo-Frost® surface cooling and intracanal cryogenic irrigation could not be determined because both interventions were evaluated as a combined protocol. Future studies have been recommended to investigate these variables independently.

## Ethical statement

Ethical approval was obtained from the Institutional Ethics Committee of Sumandeep Vidyapeeth (Deemed to be University), Vadodara, Gujarat, India (approval number: SVEIC/ON/DenHRP/Feb/25/54).

## Patient/guardian consent

Invitro study design. Not applicable for this study.

## Source(s) of support

This research was conducted under an international research collaboration between Universitas Airlangga and Universiti Teknologi Malaysia under the scheme of Airlangga Research Fund Mandatory 2023 based on the Research Collaboration Agreement Ref. No. 322/UN3.LPPM/PT.01.03/2023.

## Declaration of competing interest

The authors declare that they have no known competing financial interests or personal relationships that could have appeared to influence the work reported in this paper.
